# The effect of TRV027 on coagulation in COVID‐19: A pilot randomized, placebo‐controlled trial

**DOI:** 10.1111/bcp.15618

**Published:** 2022-12-14

**Authors:** Alexander J. Robbins, Nur Amalina Che Bakri, Edward Toke‐Bjolgerud, Aaron Edwards, Asha Vikraman, Cathy Michalsky, Michael Fossler, Nana‐Marie Lemm, Savviz Medhipour, William Budd, Athanasia Gravani, Lisa Hurley, Vikas Kapil, Aimee Jackson, Dagan Lonsdale, Victoria Latham, Michael Laffan, Neil Chapman, Nichola Cooper, Richard Szydlo, Joseph Boyle, Katrina M. Pollock, David Owen

**Affiliations:** ^1^ Imperial College Research Facility Imperial College London London UK; ^2^ Imperial College Healthcare NHS Trust London UK; ^3^ Department of Surgery and Cancer Imperial College London London UK; ^4^ Trevena, Inc. Chesterbrook Pennsylvania USA; ^5^ William Harvey Research Institute, Centre for Cardiovascular Medicine and Devices, Faculty of Medicine and Dentistry Queen Mary University London London UK; ^6^ Cancer Research Clinical Trials Unit University of Birmingham Birmingham UK; ^7^ Department of Clinical Pharmacology St George's University of London London UK; ^8^ Department of Critical Care St George's University Hospitals NHS Foundation Trust London UK; ^9^ Centre for Haematology Imperial College London London UK; ^10^ Department of Inflammation and Immunity Imperial College London London UK; ^11^ National Heart and Lung Institute Imperial College London London UK; ^12^ Department of Brain Sciences Imperial College London London UK

**Keywords:** clinical trials, coagulation, randomized controlled trial

## Abstract

COVID‐19 causes significant thrombosis and coagulopathy, with elevated D‐dimer a predictor of adverse outcome. The precise mechanism of this coagulopathy remains unclear; one hypothesis is that loss of angiotensin‐converting enzyme 2 activity during viral endocytosis leads to pro‐inflammatory angiotensin‐II accumulation, loss of angiotensin‐1‐7 and subsequent vascular endothelial activation. We undertook a double‐blind randomized, placebo‐controlled experimental medicine study to assess the effect of TRV027, a synthetic angiotensin‐1‐7 analogue on D‐dimer in 30 patients admitted to hospital with COVID‐19. The study showed a similar rate of adverse events in TRV027 and control groups. There was a numerical decrease in D‐dimer in the TRV027 group and increase in D‐dimer in the placebo group; however, this did not reach statistical significance (*P* = .15). A Bayesian analysis demonstrated that there was a 92% probability that this change represented a true drug effect.

**What is already known about this subject**

- COVID‐19 is associated with a hypercoagulable phenotype with subsequent thrombosis causing significant morbidity and mortality.
- The mechanistic basis underlying this phenotype has yet to be fully described.
- One putative mechanism is endothelial activation via angiotensin‐II signalling due to a dysregulated renin angiotensin aldosterone system (RAAS).

**What this study adds**

- This is the first trial of TRV027, an angiotensin 1‐7 analogue, in COVID‐19 seeking to ameliorate a dysfunctional RAAS.
- There was a non‐statistically significant decrease in D‐dimer in those receiving TRV027.
- This trend suggests larger studies that are required to reveal any clinically relevant effects.

## 1 INTRODUCTION

Endothelial activation and microvascular thrombosis contribute significantly to the morbidity and mortality associated with COVID‐19.^1^ Abnormal activation of the clotting cascade and endothelial injury can lead to pulmonary thrombosis, renal emboli, cerebrovascular accident and deep vein thrombosis.^1^ Infection with SARS‐CoV‐2 is associated with a significantly greater risk of venous thromboembolism and microvascular thrombosis than other inflammatory lung conditions, such as influenza.^1^, ^2^, ^3^ Patients with a raised D‐dimer, and therefore evidence of significant clotting cascade activation, have worse outcomes.^4^

SARS‐CoV‐2 enters cells by binding angiotensin‐converting enzyme 2 (ACE2). In so doing, ACE2 is internalized and its function lost.^5^, ^6^, ^7^, ^8^ In a Syrian hamster model of severe COVID‐19 disease, a pseudovirus expressing S protein led to decreased levels of ACE2 in the lung compared with mock infection.^9^ ACE2 is a key enzyme in the renin angiotensin aldosterone system (RAAS). ACE2 converts the peptide hormone angiotensin‐II (Ang‐II) to angiotensin‐(1‐7) (Ang‐(1‐7)). Ang‐(1‐7) acts as a biased agonist competing with Ang‐II to bind to the angiotensin‐II receptor type 1 (AGTR1) and engaging the ß‐arrestin pathway rather than the canonical Gq pathway.^10^ If ACE2 function is lost, the combination of both increased Ang‐II and depleted Ang‐(1‐7) would induce vascular cell activation. In animal models excessive Ang‐II and depleted Ang‐(1‐7) causes pathology similar to that seen in COVID‐19, such as myocardial micro‐infarcts, glomerular thrombosis and coagulopathy.^11^, ^12^, ^13^ This pathology was ameliorated by treatment with Ang‐(1‐7).^11^, ^12^, ^13^

We tested the hypothesis that COVID‐19‐associated coagulopathy is partly driven by the combination of increased Ang‐II activity and decreased Ang‐(1‐7) signalling. We used an Ang‐(1‐7) analogue, TRV027, to mimic Ang‐(1‐7) and antagonize Ang‐II signalling at the AGTR1. TRV027 is an eight‐amino acid polypeptide based on Ang‐(1‐7). Compared with Ang‐(1‐7), the N‐terminal aspartate is substituted for a sarcosine (a non‐natural amino acid), and there is an additional alanine residue at the C‐terminus. TRV027 has a half‐life of 4.2–15.8 min resulting in rapid achievement of steady state concentration. TRV027 was well tolerated in phase II trials in hospitalized patients with acute decompensated heart failure, with no impact on systolic blood pressure. Here we examine whether administration of TRV027 reduces D‐dimer levels, a surrogate marker of coagulopathy, in patients hospitalized with COVID‐19.

## 2 METHODS

A double‐blind randomized, placebo‐controlled experimental medicine study of TRV027 in adult patients admitted to hospital with COVID‐19 was conducted (NCT04419610).^14^ The study was approved by the London South East Research Ethics Committee and Health Research Authority (REC 20/HRA/3414) and was overseen by an independent data and safety monitoring board (DSMB).

Participants were eligible for inclusion if they were >18 years of age, screened within 96 h of a confirmed SARS‐CoV‐2 polymerase chain reaction (PCR) test, admitted to an inpatient ward and had a systolic blood pressure between 100 and 180 mmHg. Participants were excluded if they had concurrent medical conditions that were likely to significantly affect D‐dimer measurements, were receiving angiotensin receptor blockade (ARB) therapy, had medical conditions that would compromise the safety or scientific integrity of the study and were pregnant or breastfeeding. Informed consent was obtained from patients or a consultee where they lacked capacity to consent.

Enrolled participants were block randomized by age to receive 0.9% sodium chloride intravenous infusion or TRV027 at a rate of 12 mg/h. Based on previous trials with infusion rates from 1.25–31.25 mg/h and in vitro AT1R binding data, this infusion rate was predicted to give a median receptor occupancy rate in excess of 80%.^14^ The infusion was given for a maximum of 7 days or until the participant was discharged.

Blood tests were taken on Day 1, prior to the study infusion commencing, and then on Days 3, 5 and 8. The primary outcome of the study was change in D‐dimer at Day 3, that is, after at least 48 h of infusion.

Participants' heart rate, blood pressure, respiratory rate, oxygen saturations and temperature were monitored and recorded four times daily. Glasgow Coma Scale (GCS) was assessed daily and the sequential organ failure assessment score (SOFA) calculated on Days 3, 5 and 8. Participants' electronic patient records were reviewed daily for adverse events; these were classified using a modified version of the Common Terminology Criteria for Adverse Events available in the study protocol.^14^

### 2.1 Statistics

The study was reported in line with CONSORT guidelines.^15^ A sample size of 60 was required for an 80% power to detect a 30% change in D‐dimer between groups. Data from electronic medical records were recorded using a REDCap database.^16^ Continuous data were summarized by *n*, mean, median, interquartile range and range, and categorical data as number and percentage. Outcome values were compared between control and TRV027 groups using the Mann–Whitney non‐parametric test.

Due to a significant reduction in the rate of patient admissions, the DSMB recommended that recruitment was stopped at 30 patients and also recommended the use of a Bayesian analysis on the primary outcome.

### 2.2 Bayesian analysis

A Bayesian regression model with non‐informative prior was used to assess percent change in D‐Dimer levels across treatment groups. The Bayesian regression model included treatment group and age as predictors, along with an interaction term of age and treatment group. SAS version 9.4 proc genmod was utilized to execute this analysis. The final model was determined to be age, treatment and treatment by age interaction.

### 2.3 Nomenclature of targets and ligands

Key protein targets and ligands in this article are hyperlinked to corresponding entries in http://www.guidetopharmacology.org, the common portal for data from the IUPHAR/BPS Guide to PHARMACOLOGY, and are permanently archived in the Concise Guide to PHARMACOLOGY 2019/20.^17^

## 3 RESULTS

A total of 51 patients were screened for enrolment; 21 individuals were either ineligible or declined enrolment (Figure S1). Of the 30 enrolled, two further participants withdrew consent prior to commencing the infusion, one from each group. Age and sex of patients were not significantly different between TRV027 and control (Table 1). Baseline biochemical and haematology blood results are included in Table S1. In hospital, use of steroids was not statistically different, 10/10 (100%) in the TRV027 group and 8/11 (72.7%), respectively. All patients received anticoagulants; in the TRV027 group, 30% were anticoagulated therapeutically compared with 45.4% in the control. The remainder received lower dose prophylactic anticoagulation; enoxaparin was the most common agent used (80.0% *vs*. 72.7%). Only one patient in the control group was taking an antiplatelet. Other concomitant medication use is detailed in Table S2.

**TABLE 1:** Baseline demographics and disease severity in those recruited to the COVRAS trial and randomized to either control arm or TVR027

	Full analysis set		Incomplete analysis set	
	Control (*n* = 11)	TRV027 (*n* = 10)	Control (*n* = 13)	TRV027 (*n* = 15)
	Median (range) or (%)	Median (range) or (%)	Median (range) or (%)	Median (range) or (%)
**Age (years)**	72 (43–79)	70.5 (47–89)	70.0 (43–79)	67.0 (47–89)
**Age group**				
<60	4 (36.4)	2 (20.0)	6 (46.2)	6 (40.0)
60–69	0	2 (20.0)	0	2 (13.3)
>69	7 (63.6)	6 (60.0)	7 (53.8)	7 (46.7)
**Gender**				
Male	3 (27.3)	4 (40.0)	4 (30.8)	8 (53.3)
Female	8 (72.7)	6 (60.0)	9 (69.2)	7 (46.7)
**Ethnicity**				
White	2 (18.2)	4 (40.0)	2 (15.4)	5 (33.3)
Mixed	0 (0)	0 (0)	0 (0)	1 (6.7)
Asian	3 (27.3)	0 (0)	3 (23.1)	0 (0)
Black	2 (18.2)	0 (0)	2 (15.4)	0 (0)
Other	4 (36.4)	5 (50.0)	5 (38.5)	7 (46.7)
Not reported	0 (0)	1 (10.0)	1 (7.7)	2 (13.3)
**Disease severity**				
Mild	3 (27.3)	0 (0)	3 (23.1)	1 (6.7)
Moderate	0 (0)	1 (10.0)	0	1 (6.7)
Severe	5 (45.4)	1 (10.0)	7 (53.8)	5 (33.3)
Critical	3 (27.3)	8 (80.0)	3 (23.1)	8 (53.3)

*Note*: Data are presented for the full analysis set who received at least two infusions and had blood samples taken on Day 3 and the incomplete analysis set who were randomized but withdrawn before the primary endpoint.

Ten patients in the TRV027 group and 11 in the placebo group completed >48 h of study infusion and had at least one set of bloods taken at 48 h; these participants contributed to the full analysis set (FAS). Reasons for early withdrawal are included in Table S3. Drug compliance for those in the FAS until the Day 3 primary end point is summarized in Table S4. Briefly two participants in each group had a pause of >6 h in their infusion, and all were subsequently restarted.

Most patients had severe COVID‐19, as defined by the World Health Organization COVID‐19 Disease Severity Classification at screening, with 8/10 (80%) in the TRV027 arm and 5/11 (46%) in the control arm. The proportion with critical COVID‐19 was greater in the control group, 3/11 (27%), while there were no patients in the TRV027 group with critical COVID‐19 at screening (Table 1).

### 3.1 Venous blood assessments

In those who received TRV027, the median baseline D‐dimer (802 ng/mL) decreased by 129 ng/mL between Day 1 and Day 3. In contrast, those in the control group showed a median increase of 96 ng/mL from a baseline of 945 ng/mL (Table 2 and Figure S2). This difference was not statistically significant (*P* = .15). Subsequent Bayesian analysis indicated a 92% probability of a true treatment effect for reduction in D‐dimer favouring TRV027 over control (Figure S3). There were no statistically significant changes in 12 haematological or biochemical markers of renal, liver or cardiac pathology including creatinine, bilirubin or troponin. There was a greater numerical decrease in fibrinogen levels in the control group (−0.94 g/L *vs*. − 0.67 g/L) which was not statistically significant (*P* = .97), with no difference in activated partial thromboplastin time or international normalized ratio (INR) (Table 2). Brain natriuretic peptide (BNP) was higher in those exposed to TRV027 relative to placebo (*p* = .028) (Table 2).

**TABLE 2:** Biochemical and clotting results from control and TRV027 arms with difference between baseline value (Day 1) and primary endpoint (Day 3) calculated

	Control (*n* = 11)		TRV027 (*n* = 10)		
	*n*	Difference D3 – D1	*n*	Difference D3 – D1	*P*‐value
D‐dimer	8	96	10	−129	0.15
Platelets (×10^9^/L)	10	28.5	10	25	0.82
aPTT (s)	8	−0.5	9	−1.3	0.67
Fibrinogen	9	−0.94	9	−0.67	0.97
Total bilirubin	9	0	10	−0.5	0.68
LDH	7	2	8	−21.5	0.56
Haptoglobin	5	0.13	3	0	0.13
Creatinine (umol/L)	9	−5	10	−4	0.97
BNP	6	−23.5	5	98	0.028
Troponin	6	−2.5	6	−2.5	0.51
Ferritin	8	−14	10	52.5	0.48
Pro‐calcitonin	7	0	5	0	0.57
Glucose (mmol/L)	6	0	5	−1.1	0.52

In the control group, there were three serious adverse events: death from severe COVID‐19 pneumonia, bacterial sepsis and pulmonary embolism (Tables 3, S5 and S6). Two non‐serious adverse events were recorded: mild transient hypotension and a mild infusion site reaction.

**TABLE 3:** Adverse events recorded in both the control and TRV027 group categorized by seriousness, graded for severity and causal relationship to study drug

	Control		TRV027	
	No. of events	No. of subjects	No. of events	No. of subjects
Number of serious adverse events	3	3	5	4
**Reasons for SAE**				
Resulted in death	1	1	2	2
Life threatening	2	2	1	1
Congenital anomaly/birth defect	0	0	0	0
Persistent or significant	0	0	0	0
Disability/incapacity	0	0	0	0
Prolongation of hospitalization	0	0	2	2
Other	0	0	0	0
**Severity**				
Mild	0	0	0	0
Moderate	0	0	0	0
Severe	1	1	3	2
Life threatening	1	1	0	0
Death	1	1	2	2
**Causal relationship to study drug**				
Definitely	0	0	0	0
Probably	0	0	0	0
Possibly	0	0	0	0
Unlikely	0	0	0	0
Not related	3	3	5	4
Not assessable	0	0	0	0

In the TRV027 group, there were five serious adverse events (Tables 3, S5 and S6). Two patients developed progressive respiratory failure and died of severe COVID‐19 pneumonia. One patient was transferred to intensive care and developed hypotension following sedation and intubation; they were subsequently diagnosed with a pulmonary embolism. One patient developed confusion coinciding with dexamethasone use, a steroid associated with drug‐induced psychosis. All serious adverse reactions were judged to be unrelated to TRV027 in the opinion of the investigators. One non‐serious adverse event of epistaxis was judged possibly related to the infusion.

Physiological observations were captured throughout the duration of the infusion and demonstrated no clinically significant differences between control and TRV027 arms.

## 4 DISCUSSION

In this randomized controlled experimental medicine study, we tested the hypothesis that COVID‐19‐associated coagulopathy is partly driven by RAAS dysregulation by measuring the response on D‐dimer of a biased agonist at the AGTR1 in hospitalized COVID‐19 patients. The frequentist analysis did not reach significance although the early truncation of the trial for pragmatic reasons underpowered this analysis. The Bayesian analysis suggested a 92% probability that the reduction in D‐dimer in the TRV027 group represented a true drug effect. Fibrinogen levels decreased in both groups, suggesting a consumptive process. Although this decrease was numerically greater in the control group, it was not statistically significant. There was no difference in APTT or INR suggesting that the coagulation cascade does not account for variation in fibrinogen consumption or D‐dimer production and endothelial status may be responsible. Although there was an increase in BNP, which was not corrected for multiple testing due to the small sample size, it is of unclear statistical and clinical significance. There was no demonstrable induction of hypotension during TRV027 infusion compared with placebo and no hypotensive or cardiovascular events attributed to TRV027. It does raise the possibility that augmentation of AGTR1 signalling may affect cardiovascular stress signalling.

The study infusion was well tolerated without evidence of adverse physiological effects. The rate of adverse events was similar between placebo and TRV027, as has previously been demonstrated in a study examining TRV027 in an acutely unwell population (acute decompensated heart failure).^18^

We hypothesize that SARS‐CoV2‐induced ACE2 downregulation or disruption at any site could lead to endothelial dysfunction by disrupting the endocrine Ang‐(1‐7)/Ang‐II ratio. Given that recent evidence points to an absence of meaningful endothelial ACE2 expression or direct infection of endothelium by SARS‐CoV2,^19^, ^20^ we hypothesize that ACE2 downregulation, likely in the lung, results in localized RAAS dysregulation with accumulation of Ang‐II and loss of Ang‐(1‐7) leading to local endothelial cell toxicity. This could be rescued by the use of an Ang‐(1‐7) analogue such as TRV027, through the promotion of a biased agonist effect at the AGTR1, blocking the action of Ang‐II and replacing Ang‐(1‐7).

## 5 LIMITATIONS

This study was concluded prematurely due to a reduction in local cases and therefore the results should be treated as exploratory. This study was also carried out in the United Kingdom when alpha was the predominant COVID‐19 variant. Omicron is now the dominant variant circulating globally, and the degree to which these findings are applicable should be interpreted in the context of a subtly different pathogen, albeit one that also uses ACE2 for cellular entry and is likely to cause severe disease through shared biochemical pathways.

There was significant heterogeneity in the disease severity at the time of recruitment, and patients also presented to hospital at variable stages in their disease progression. Given the small sample size, this increases the risk that our results are the result of random variation between the two groups.

Predicted AGTR1 receptor occupancy was calculated from in vitro experiments and previous clinical trial data.^14^ It is unknown if the RAAS disruption seen in COVID‐19 will lead to an up‐ or downregulation of AGTR1, effects that may theoretically alter sensitivity to TRV027.

## 6 CONCLUSION

In patients hospitalized with COVID‐19, exposure to TRV027 was associated with a trend towards reduction in D‐dimer which did not achieve statistical significance.

## COMPETING INTERESTS

K.P. and D.O. hold advisory positions on the Accelerating COVID‐19 therapeutic interventions and vaccines (ACTIV)‐4D) host tissue protocol development board, National Institute for Health, USA and on the Randomized, Embedded, Multifactorial Adaptive Platform trial for Community‐Acquired Pneumonia (REMAP‐CAP) ACE2 renin angiotensin system (RAS) domain protocol development board. C.M. and M.J.F. are employees of and hold stock in Trevena. All other authors declare no conflicts of interest.

## CONTRIBUTORS

D.O., K.P., J.B., N.C., N.C. and M.L. conceived of and designed the study; A.R., N.B., A.E., S.M. and N.M.L. the consented participants; E.T., W.B. and A.V. collected the data. V.K., D.L. and A.J. were members of the DSMB. V.L. organized and provided pharmacy support. R.S., C.M. and M.F. provided statistical guidance and analysed results. A.G. provided monitoring support and L.H. was the trial manager. A.R. wrote the manuscript, and all authors were involved in its critical review.

## Supporting information


**Figure S1.** Flow diagram demonstrating screening, enrolment and completion of the trial to the primary endpoint
**Figure S2**. Scatter graph demonstrating the absolute change from baseline D‐dimer in those treated with TRV027 and those in the group
**Figure S3**. Probability distribution showing the results of the Bayesian analysis performed with non‐informative priors**Table S1.** Baseline biochemical and haematology blood tests for participants in the full analysis set for TRV027 and control groups on Day 1 bloods prior to starting infusion
**Table S2.** Baseline concomitant medication use in the complete analysis set
**Table S3.** Summary of the reasons for withdrawal from study prior to blood sampling at day 3 timepoint
**Table S4.** All instances of interruptions to infusions >6 h and reasons for these interruptions in those participants included in the full analysis set
**Table S5.** Description of serious adverse events, causality and outcomes
**Table S6.** All adverse events with higher order classification and MEDRA codes for the incomplete analysis set

## Data Availability

Individual participant data are available on reasonable request where research proposals have ethical approval. Participant data will be anonymized and only available for participants consenting to data sharing.
