# Supplementary material for: The effect of TRV027 on coagulation in COVID‐19: A pilot randomized, placebo‐controlled trial
Source: Br J Clin Pharmacol. 2022 Dec 14;89(4):1495–501. doi: 10.1111/bcp.15618 (PMC10952550; doi:10.1111/bcp.15618)
Supplement: Supplementary file 1 — Figure S1. Flow diagram demonstrating screening, enrolment and completion of the trial to the primary endpoint Figure S2. Scatter graph demonstrating the absolute change from baseline D‐dimer in those treated with TRV027 and those in the group Figure S3. Probability distribution showing the results of the Bayesian analysis performed with non‐informative priorsTable S1. Baseline biochemical and haematology blood tests for participants in the full analysis set for TRV027 and control groups on Day 1 bloods prior to starting infusion Table S2. Baseline concomitant medication use in the complete analysis set Table S3. Summary of the reasons for withdrawal from study prior to blood sampling at day 3 timepoint Table S4. All instances of interruptions to infusions >6 h and reasons for these interruptions in those participants included in the full analysis set Table S5. Description of serious adverse events, causality and outcomes Table S6. All adverse events with higher order classification and MEDRA codes for the incomplete analysis set [file BCP-89-1495-s001.docx]

**Supplemental Data: The effect of TRV027 on coagulation in COVID 19: A Pilot Randomised Controlled Trial**

**Full Structure of Angiotensin 1-7 and TRV027**

**Ang-(1-7):**

H-Asp-Arg-Val-Tyr-Ile-His-Pro-OH

**TRV027:**

Sar-Arg-Val-Tyr-Ile-His-Pro-D-Ala-OH

**Bayesian Model Form:**

The mathematical form of this model is:

*Y_i_ = ß_1_ x_i,1_ + ß_2_ x_i,2_ + ß_3_ x_i,3_ + ß_4_ x_i,4_ + ß_n_ x_i,n_*

*= ß_1_ + ß_2_ × Group_i_ + ß_3_ × age_i_ + ß_4_ × Group_i_ × age_i_*

*x_i,1_ =* 1 for each subject *i*

*x_i,2_ =* 0 if subject *i* is on the control treatment, 1 if on TRV027 treatment

*x_i,3_ = age* of the subject *i*

*x_i,4_* = *x_i,2_ × x_i,3_* interaction term of treatment and age

*ß_n_ x_i,n_* = additional covariates

Other covariates explored included baseline SOFA score, baseline disease severity, prior medication use of steroids (Dexamethasone or Hydrocortisone), and use of Remdesivir.

**
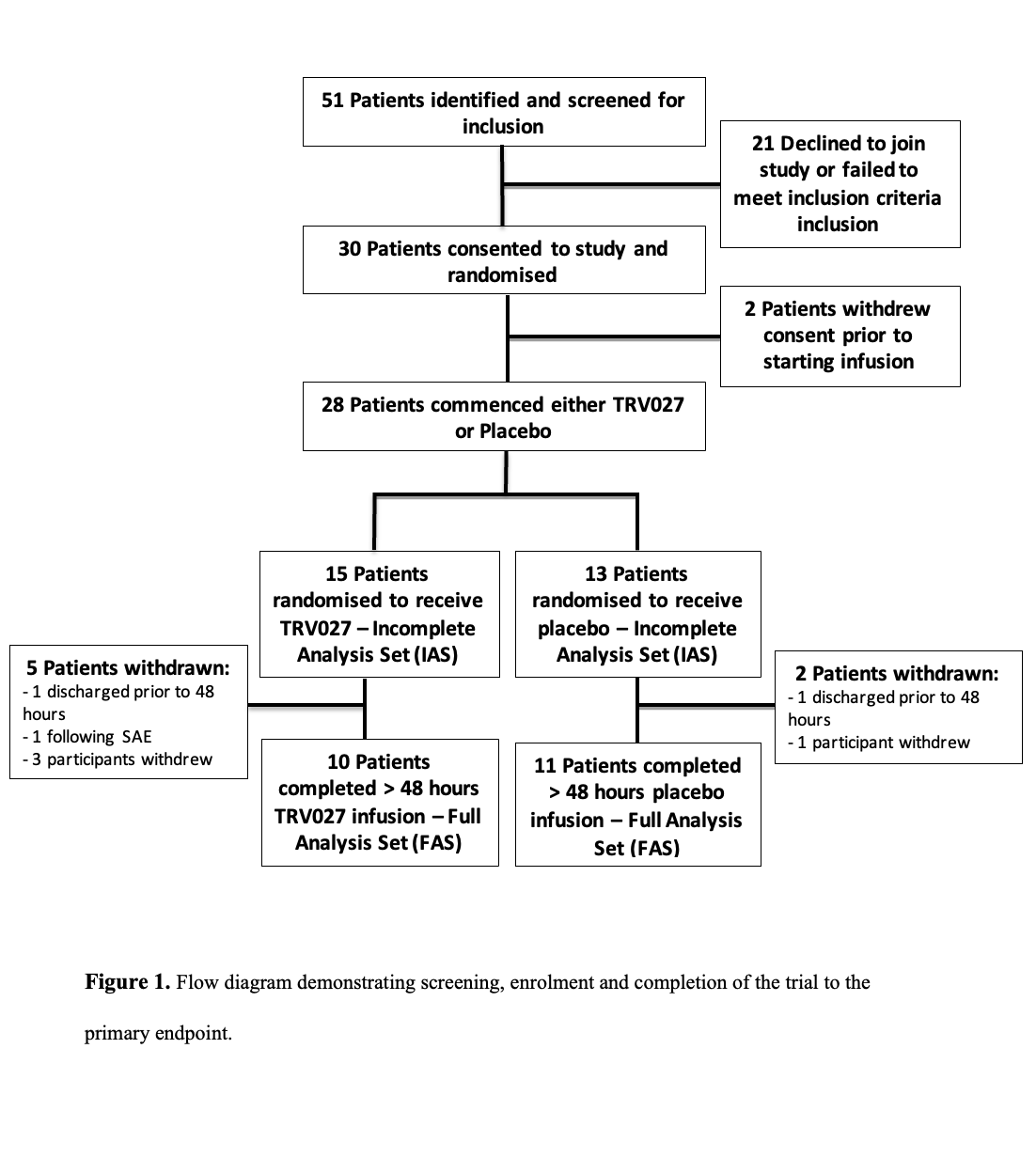
**

**
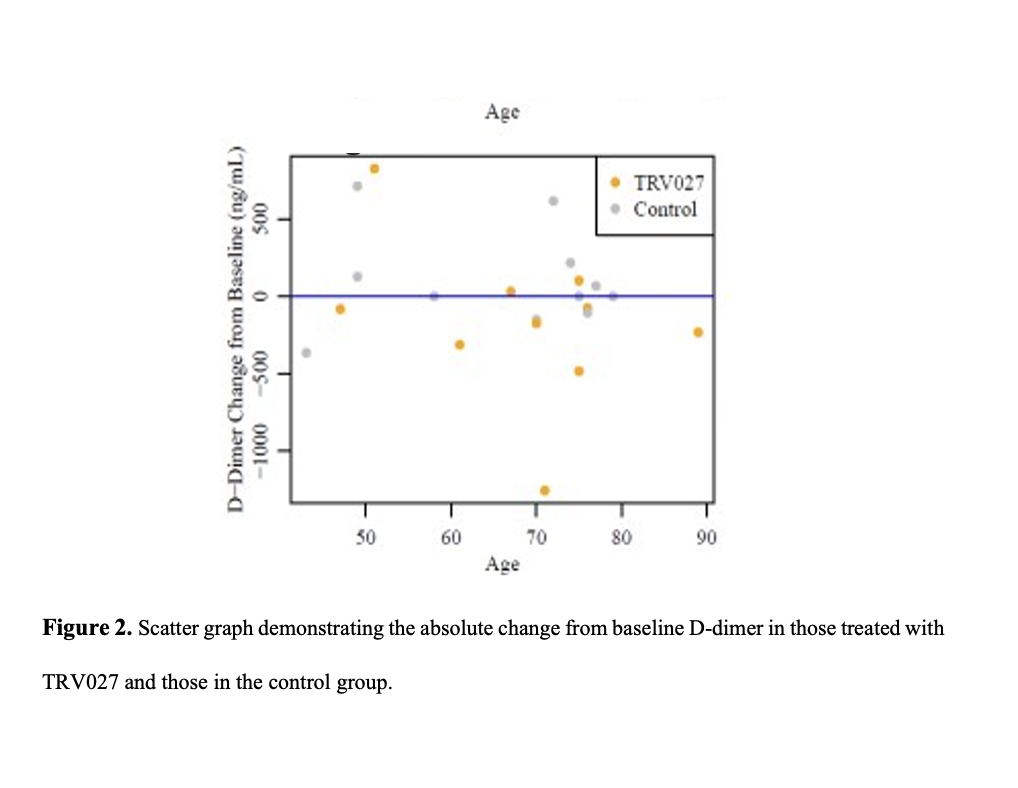
**

**
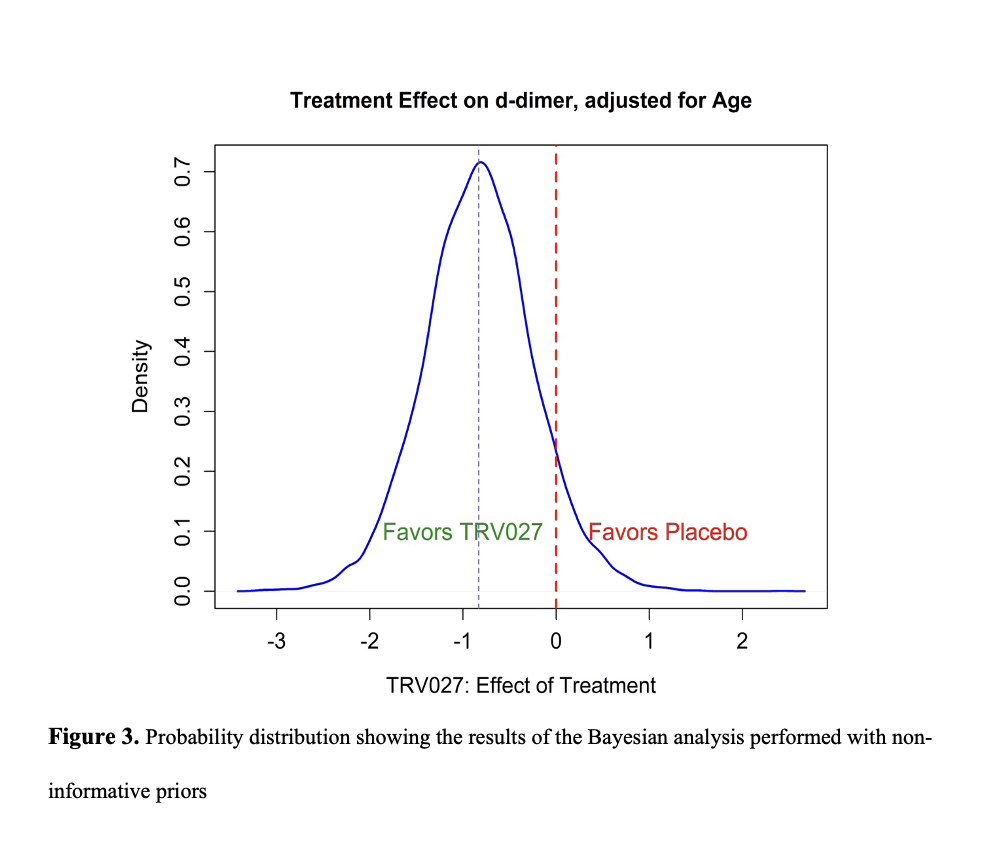
**

| **Blood Result** | **TRV027**  **Median (IQR)** | **Control**  **Median (IQR)** |  |
| --- | --- | --- | --- |
| **APTT (Seconds)** | 31.2 (27.2 - 32.1) | 31.3 (28.0 - 32.7) |  |
|  |  |  |  |
| **Brain Natriuretic Peptide** | 74.0 (46.0 - 155.0) | 42.5 (36.0 - 77.0) |  |
|  |  |  |  |
| **Creatinine** | 68 (61 - 90) | 64 (54 - 79) |  |
|  |  |  |  |
| **D-Dimer** | 802 (555 - 1357) | 945 (787 - 1230) |  |
|  |  |  |  |
| **Ferritin** | 12 (7 - 18) | 6 (5 - 11) |  |
|  |  |  |  |
| **Fibrinogen** | 5.15 (4.32 - 6.49) | 5.20 (4.21 - 5.37) |  |
|  |  |  |  |
| **Glucose** | 7.4 (6.5 - 9.2) | 6.9 (5.5 - 13.0) |  |
|  |  |  |  |
| **Haptoglobin** | 3.67 (2.91 - 3.67) | 3.40 (2.20 - 3.67) |  |
|  |  |  |  |
| **International Normalised**  **Ratio (INR)** | 1.1 (1.0 - 1.1) | 1.0 (1.0 - 1.1) |  |
|  |  |  |  |
| **Lactate Dehydrogenase** | 316 (307 - 503) | 416 (289 - 551) |  |
|  |  |  |  |
| **Platelet Count** | 261 (194 - 386) | 189 (162 - 268) |  |
|  |  |  | |
| **Procalcitonin** | 0.08 (0.06 – 0.17) | 0.06 (0.03 – 0.12) | |
|  |  |  | |
| **Renin** | 1.1 (0.6 - 1.7) | 1.2 (0.5 - 2.9) | |
|  |  |  | |
| **Bilirubin** | 8 (8 - 9) | 10 (5 - 10) | |
|  |  |  | |
| **Troponin** | 689 (475 - 2071) | 344 (154 - 1427) | |
|  |  |  | |

**Supplemental Table 1.** Baseline biochemical and haematology blood tests for participants in the Full Analysis Set for TRV027 and control groups on Day 1 bloods prior to starting infusion.

| **Medication​** | **TRV027​ (N = 10)​ N/(%)** | **Placebo​ (N = 11)​ N/(%)** |
| --- | --- | --- |
| **Anticoagulants**  Apixaban​​  Enoxaparin​  Rivaroxaban​  ​ | ​  2 (20.0%)​  8 (80%)  0 (0.0%) | ​  1 (9.1%)  8 (72.7%)​  2 (18.2%)​ |
| **Anticoagulation Regime** |  |  |
| Therapeutic  Prophylactic | 3 (30.0%)  7 (70.0%) | 5 (45.5%)  6 (54.5%) |
| **Glucocorticoids**  Dexamethasone  Dexamethasone/Prednisolone  Hydrocortisone | 9 (90.0%)  1 (10.0%)  0 (0.0%) | 7 (63.6%)  0 (0.0%)  1 (9.1%) |
| **IL-6 Blockade**  Tocilizumab  Sarulimab | 2 (20.0%)  1 (10.0%) | 2 (18.2%)  1 (9.1%) |
| **Antiplatelet**  Aspirin | 0 (0.0%) | 1 (9.1%) |
| **Antivirals**  Remdesivir | 5 (50.0%) | 7 (63.6%) |

**Supplemental Table 2.** Baseline concomitant medication use in the Complete Analysis Set

**Reasons for withdrawal prior to Day 3 timepoint**

Within the TRV027 group three participants withdrew consent prior to having blood tests at 48 hours (Figure 1). The most common reason for withdrawal was cited as inconvenience of the continuous infusion and a wish to avoid intravenous cannulation. One participant was withdrawn from the study on discussion with the treating clinical team after developing acute confusion. This was assessed by the investigator as unrelated to the infusion. The study infusion in one participant was discontinued as they were well enough to be discharged within 48 hours of admission. Within the control group, one participant withdrew consent prior to blood tests at 48 hours, and one participant was well enough to be discharged after 24 hours.

| **Reason for withdrawal** | **TRV027** | **Control** |
| --- | --- | --- |
| Patient withdrew consent | 3 | 1 |
| Unrelated Adverse Event | 1 | 0 |
| Discharged after < 48 hours | 1 | 1 |

**Supplemental Table 3.** Summary of the reasons for withdrawal from study prior to blood sampling at day 3 timepoint

| **Participant** | **Allocation** | **Interruption > 6 hours** | **Reason for infusion interruption** |
| --- | --- | --- | --- |
| 402-8 | Placebo | Yes | Extravasation at cannulation site |
| 402-19 | TRV027 | Yes | Unable to obtain IV access |
| 402-20 | Placebo | Yes | Paused on admission to Intensive Care |
| 402-22 | TRV027 | Yes | Paused on admission to Intensive Care |

**Supplementary Table 4.** All instances of interruptions to infusions >6 hours and reasons for these interruptions in those participants included in the Full Analysis Set.

| **Participant** | **Allocation** | **Serious Adverse Event** | **Causality** | **MEDRA Code** | **Outcome** |
| --- | --- | --- | --- | --- | --- |
| 402-3 | TRV027 | Severe COVID-19 pneumonia | Unrelated | 10084380 | Death |
| 402-5 | Placebo | Severe COVID-19 pneumonia | Unrelated | 10084380 | Death |
| 402-7 | TRV027 | Confusion | Unrelated | 10010300 | Resolved |
| 402-11 | TRV027 | Severe COVID-19 pneumonia | Unrelated | 10084380 | Death |
| 402-25 | Placebo | Pulmonary Embolism | Unrelated | 10037377 | Resolved |
| 402-28 | Placebo | Bacterial Sepsis | Unrelated | 10053840 | Resolved |
| 402-30 | TRV027 | Hypotension | Unrelated | 10021097 | Resolved |
| 402-30 | TRV027 | Pulmonary Embolism | Unrelated | 10037377 | Ongoing long term follow up |

**Supplemental Table 5.** Description of Serious adverse events, causality and outcomes

| **Classification​** | **Adverse Event Verbatim Term​** | **MEDRA Code** | **TVR027 N=15**  **N(%)** | **Placebo​N=13​**  **N (%)​** |
| --- | --- | --- | --- | --- |
| **Infection​** | Severe COVID-19 Pneumonia  Bacterial sepsis​ | 10084380  10053840 | 2 (13.4%)​  0 (0.0%)​ | 1 (7.7%)​  1 (7.7%)​ |
| **Epistaxis​** | Epistaxis​ | 10015090 | 1 (6.7%)​ | 0 (0.0%)​ |
| **Hypotension​** | Hypotension​ | 10021097 | 1 (6.7%)​  ​ | 1 (7.7%)​ |
| **Pulmonary embolism​** | Pulmonary embolism​ | 10037377 | 1 (6.7%)​ | 1 (7.7%)​ |
| **Confusion​** | Confusion​ | 10010300 | 1 (6.7%)​ | 0 (0.0%)​ |
| **Injection Site Reaction​** | Injection site reaction​ | 10022095 | 0 (0.0%)​ | 1 (7.7%)​ |

**Supplemental Table 6.** All adverse events with higher order classification and MEDRA codes for the Incomplete Analysis Set.
